# New Insights into the Chloroplast Outer Membrane Proteome and Associated Targeting Pathways

**DOI:** 10.3390/ijms23031571

**Published:** 2022-01-29

**Authors:** Michael Fish, Delaney Nash, Alexandru German, Alyssa Overton, Masoud Jelokhani-Niaraki, Simon D. X. Chuong, Matthew D. Smith

**Affiliations:** 1 Department of Biology, Wilfrid Laurier University, Waterloo, ON N2L 3C5, Canada; fish1960@mylaurier.ca (M.F.); germ2740@mylaurier.ca (A.G.); 2 Department of Biology, University of Waterloo, Waterloo, ON N2L 3G1, Canada; d2nash@uwaterloo.ca (D.N.); akoverton@uwaterloo.ca (A.O.); schuong@uwaterloo.ca (S.D.X.C.); 3 Department of Chemistry & Biochemistry, Wilfrid Laurier University, Waterloo, ON N2L 3C5, Canada; mjelokhani@wlu.ca

**Keywords:** chloroplast-targeting pathways, chloroplast outer membrane proteome, signal anchored protein, tail anchored protein, β-barrel protein, β-signal, chloroplast transit peptide, TOC complex, AKR2, OEP80

## Abstract

Plastids are a dynamic class of organelle in plant cells that arose from an ancient cyanobacterial endosymbiont. Over the course of evolution, most genes encoding plastid proteins were transferred to the nuclear genome. In parallel, eukaryotic cells evolved a series of targeting pathways and complex proteinaceous machinery at the plastid surface to direct these proteins back to their target organelle. Chloroplasts are the most well-characterized plastids, responsible for photosynthesis and other important metabolic functions. The biogenesis and function of chloroplasts rely heavily on the fidelity of intracellular protein trafficking pathways. Therefore, understanding these pathways and their regulation is essential. Furthermore, the chloroplast outer membrane proteome remains relatively uncharted territory in our understanding of protein targeting. Many key players in the cytosol, receptors at the organelle surface, and insertases that facilitate insertion into the chloroplast outer membrane remain elusive for this group of proteins. In this review, we summarize recent advances in the understanding of well-characterized chloroplast outer membrane protein targeting pathways as well as provide new insights into novel targeting signals and pathways more recently identified using a bioinformatic approach. As a result of our analyses, we expand the known number of chloroplast outer membrane proteins from 117 to 138.

## 1. Introduction

The compartmentalization of eukaryotic cells by membrane-bound organelles provides diverse environments for a wide variety of biochemical pathways essential to cell function and survival. Plastids are a dynamic class of organelle that evolved from an ancient cyanobacterial endosymbiont [1,2,3]. As they evolved, plastids adopted various central functions in cell metabolism and biosynthesis as well as in signalling, embryogenesis, leaf development, gravitropism, temperature response, and plant–microbe interactions [4]. Unlike other organelles, plastids can differentiate into various types from a common precursor, known as the proplastid, which serve different metabolic needs throughout plant tissues [5,6,7,8]. Plastids can also transition between types in response to different developmental [9,10] or environmental cues [11,12,13,14,15]. One such example is the process of photomorphogenesis, where, in the presence of light, the etioplasts of leaf cells grown in the dark are converted to green photosynthetic chloroplasts, the most notable and well-characterized type of plastids [16]. The ability of plants to trigger these types of transitions and maintain a variety of plastid types in different tissues and at different stages of life is what allowed for the radiant expansion of the plant kingdom [6]. The interconversion of plastids is made possible by the coordinated remodelling of the plastid proteome [17,18,19].

Over the course of evolution, a large majority of plastid genes were transferred to the nuclear genome by horizontal gene transfer [1,20,21,22,23]. As a result, plastid biogenesis and function rely on the fidelity of intracellular protein trafficking pathways to deliver the corresponding proteins to plastids [24]. To recognize and import chloroplast precursor proteins synthesized in the cytosol, plant cells evolved complex proteinaceous machinery at the outer and inner membranes of the chloroplast known as the general import apparatus, composed of the translocon at the outer membrane of the chloroplast (TOC complex) and the translocon at the inner membrane of the chloroplast (TIC complex) [1,25]. Chloroplast outer membrane proteins, including components of the TOC complex, use alternative targeting pathways to reach the chloroplast outer membrane [26]. A unique set of challenges exist for membrane proteins that are targeted to their resident membranes post-translationally. As the hydrophobic segments of their amino acid chains are synthesized on cytosolic ribosomes, chaperones are often required to maintain stability, prevent misfolding and avoid aggregation before the proteins reach their target membranes [27]. In some cases, the chaperones themselves serve as targeting elements whereas in other cases, a targeting sequence may engage a receptor at the target membrane surface or local secondary and tertiary structures may engage the membrane directly, inducing self-insertion [28].

Classifying common targeting pathways is challenging, as the specific mechanisms used by many outer membrane proteins remain uncharacterized. This is, in large part, due to the limited number of known and confirmed chloroplast outer membrane proteins, as well as the difficulties associated with studying membrane proteins in vitro [29]. It is even the case that some proteins use a combination of redundant strategies [26], increasing the complexity of distinguishing between these mechanisms further. Advances in proteomics and the development of more powerful bioinformatic tools have led to the identification and characterization of an increasing number of chloroplast outer membrane proteins in recent years [29,30]. In this review, we discuss current advances in our understanding of the targeting signals and pathways used by chloroplast outer membrane proteins during their biogenesis. Further, we put forth a proteome-wide bioinformatic approach for identifying novel chloroplast outer membrane protein-targeting signals and pathways. This analysis allowed us to expand the current list of chloroplast outer membrane proteins from 117 to 138.

## 2. The Chloroplast Outer Membrane

### 2.1. Composition and Function of the Chloroplast Outer Membrane

Like their bacterial ancestors and mitochondria, the other endosymbiotic organelles found in eukaryotes, chloroplasts are surrounded by two membranes that differ in function and composition [31]. The inner membrane is studded with transport proteins and tightly regulates the flux of ions and metabolites between the intermembrane space (IMS) and the stroma, the interior compartment of the chloroplast [32]. The inner membrane is composed primarily of galactolipids with some phospholipids and sulfolipids [33]. In contrast, the chloroplast outer membrane is permeable to ions and metabolites and controls the recognition and import/insertion of chloroplast proteins [32]. It also serves as the site for galactolipid biosynthesis [34] and is composed primarily of phospholipids and galactolipids in equal proportions with some sulfolipids [33]. The entire chloroplast outer membrane proteome is encoded by genes in the nucleus, synthesized on cytosolic ribosomes, and targeted post-translationally [20,21,22]. Therefore, these proteins must contain signals that direct them to the chloroplast outer membrane. Inoue (2015) published a comprehensive list of chloroplast outer membrane proteins in *Arabidopsis thaliana*, of which there are 117, and categorized them based on their function [30]. This represents a diverse proteome with functions including: solute and ion transport, protein import, protein turnover and modification, lipid metabolism, carbohydrate metabolism and regulation, other metabolism and regulation and intracellular communication, as well as proteins of unknown function [30]. More proteins still have been identified that associate with the chloroplast outer membrane surface but are not inserted in the membrane [35]. This functional diversity highlights the crucial role that the chloroplast outer membrane plays in plastid biogenesis, cell metabolism and intracellular signalling between plastids and the rest of the cell. Despite the advances made in our understanding of chloroplast precursor protein import into the stroma, the targeting and insertion mechanisms for chloroplast outer membrane proteins remains elusive. This gap is further exaggerated when compared to our understanding of mitochondrial outer membrane proteins [36].

### 2.2. Topologies of Chloroplast Outer Membrane Proteins

Based on their structure and topology, chloroplast outer membrane proteins fit into several categories. α-helical proteins are categorized by the number and location of their transmembrane domain(s) (TMD(s)). Signal anchored (SA) proteins contain single membrane-spanning α-helices located at their N-terminus and tail anchored (TA) proteins contain single membrane-spanning α-helices located at their C-terminus [37]. Both SA and TA proteins adopt similar topologies in which their soluble domains are exposed to the cytosol, with some containing short extensions into the IMS. Although very few have been characterized, some α-helical proteins are anchored by a single membrane-spanning α-helix in the middle of their sequences, containing both N- and C-terminal soluble domains exposed at opposite sides of the membrane. These are not classified as SA or TA proteins. Further, some α-helical proteins in the chloroplast outer membrane contain two or more membrane-spanning α-helices. Finally, β-barrel proteins span the membrane through the formation of a cylindrical barrel composed of β-strands [27,38]. Beyond these well-established categories of integral membrane proteins are proteins that contain either or both α-helices and β-strands, which form uncharacterized membrane anchors, proteins that are anchored to the membrane by the covalent attachment to lipid molecules, and peripheral membrane proteins that rely on electrostatic or hydrophobic interactions at the membrane surface, as well as on interactions with integral membrane proteins [27,39]. 

## 3. Protein Entry into Chloroplasts: Structure and Function of the TOC Complex

The mechanisms by which chloroplast precursor proteins are targeted to the chloroplast, recognized, and imported to the stroma by the general import apparatus have been extensively reviewed [25,40,41,42,43]. In brief, chloroplast precursor proteins are synthesized on cytosolic ribosomes and targeted to the chloroplast post-translationally via a cleavable N-terminal transit peptide (TP) [25,44,45,46]. TP sequences are moderately hydrophobic, containing an amphipathic α-helix. They are typically rich in hydroxylated and basic residues, void of acidic residues and often contain multiple proline residues [46,47,48,49,50]. A lack of arginine residues in TPs differentiate them from mitochondrial presequences and preclude mitochondrial targeting [51]. Molecular chaperones in the cytosol guide precursor proteins to the chloroplast surface in an import competent state [52]. Heat shock proteins (Hsp) 70 and 90 are involved in the transport of most chloroplast precursor proteins containing TPs [53] and may use TOC64 as a receptor [54,55]. Hsp70 forms a guidance complex with the 14-3-3 protein, which interacts directly with the TOC complex [52,53]. The TOC complex mediates recognition and import of nuclear-encoded precursor proteins into the chloroplast through the TOC-TIC supercomplex, after which the TP is cleaved in the stroma by a stromal processing peptidase [56,57]. 

The components of the core TOC complex were originally identified in pea (*Pisum sativum*) and *Arabidopsis thaliana* [58], although its composition and function appear to be highly conserved across plant species [59,60]. TOC34 and TOC159 GTPase receptors act to recognize the cleavable N-terminal TPs of chloroplast precursor proteins before they are translocated across the outer membrane via the TOC75 translocation channel [61]. Cryo-electron microscopy was used to shed light on the organization of the TOC complex and its subunits [62]. This, in combination with affinity purification and electrophoretic techniques, suggest the TOC complex exists in a 4:4:1 (TOC75:TOC34:TOC159) arrangement [62,63,64,65]. Further, paralogous TOC complexes have been identified in *Arabidopsis thaliana*, which may be responsible for the recognition and import of different subsets of chloroplast precursor proteins (housekeeping vs. photosynthetic) [5,17,18,19]. Functionally distinct TOC complexes are defined by the presence of TOC34 (TOC33) and TOC159 (TOC90, TOC120, TOC132) receptor homologs, where TOC75 is central to all TOC complexes [66]. The abundance and ratio of various paralogous TOC complexes in the plastid membrane are likely to play a role in plastid biogenesis and the transition of plastids between various types based on developmental and environmental signals [9], such as in the process of photomorphogenesis described previously. 

Less understood is the proteinaceous machinery present in the chloroplast outer membrane that is responsible for the recognition and insertion of α-helical and β-barrel chloroplast outer membrane proteins. In mitochondria, the translocon at the outer membrane of the mitochondrion (TOM) complex imports β-barrel proteins, and they are then transferred to the sorting and assembly machinery (SAM) complex by IMS chaperones before being integrated into the membrane [67,68]. Most, if not all, α-helical outer membrane proteins of mitochondria are inserted directly via the mitochondrial import (MIM) complex [67]. Such distinct pathways have not been identified for chloroplast outer membrane proteins to date. 

## 4. Biogenesis of α-Helical Chloroplast Outer Membrane Proteins

### 4.1. Signal Anchored (SA) Proteins

SA and TA proteins represent a diverse array of functions, acting as receptors in pathways of protein translocation, membrane fusion, vesicle trafficking, electron transport, apoptosis and protein quality control [39,69]. SA proteins lack a cleavable TP and are anchored in the membrane by a single hydrophobic α-helix of approximately 20 amino acid residues in length at their N-terminus [30]. This TMD is flanked by a C-terminal positively charged region (CPR) and, together, the TMD and CPR act as an intrinsic targeting signal [70]. At the sequence level, it is difficult to identify a conserved sequence motif that may direct targeting. SA proteins do not share sequence similarity at their N-termini the way they do at their C-termini. Instead, proteinaceous factors in the cytosol may recognize SA proteins directed to chloroplasts and mitochondria based on their physicochemical characteristics, which are highly conserved [27]. Specifically, the majority of TMDs within SA proteins of chloroplasts and mitochondria are only moderately hydrophobic relative to SA proteins directed to the endoplasmic reticulum, with hydrophobicity values below 0.4 on the Wimley and White scale [70]. Increasing the hydrophobicity of the TMD will redirect a chloroplast SA protein to the plasma membrane [71]. Additionally, the CPR contains three or more positively charged amino acid residues that assist in the evasion of the signal recognition particle (SRP) that directs SA proteins to the ER [27]. Like the TMD, substitutions that reduce the positive charge of the CPR redirect targeting of chloroplast SA proteins to the plasma membrane [70].

**Figure 1:**
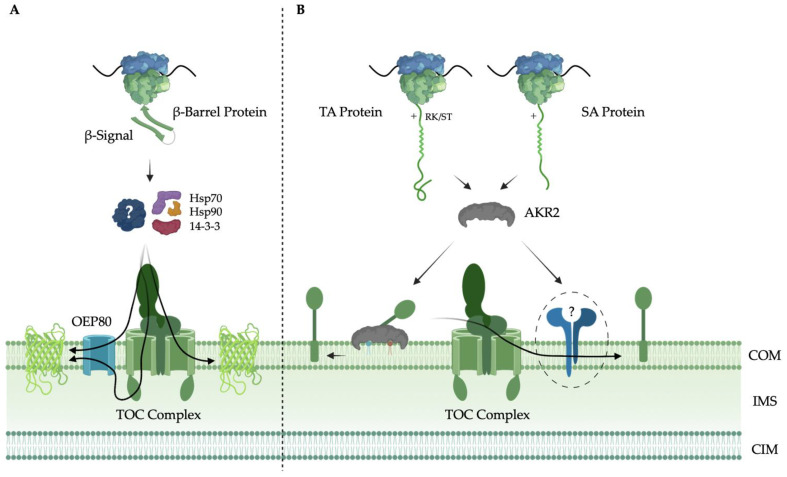
Signal anchor (SA), tail anchor (TA) and β-Barrel Mediated Targeting Pathways to the Chloroplast Outer Membrane. (**A**) β-barrel proteins are targeted to the chloroplast outer membrane by a variety of signals, like the β-signal. Although some may use Hsp70, Hsp90 and 14-3-3 proteins, a specific cytosolic chaperone that aids in their targeting is yet to be discovered. OEP80 likely plays a role in the insertion of β-barrels, although the translocon at the outer membrane of the chloroplast (TOC complex) is also involved in their targeting; (**B**) SA and TA proteins are targeted to the chloroplast outer membrane by their moderately hydrophobic N-terminal and C-terminal transmembrane α-helix, respectively, and a C-terminal positively charged region, sometimes accompanied by an RK/ST motif for TA proteins. Both are guided by ankyrin repeat-containing protein 2 (AKR2), which interacts with monogalactosyldiacylglycerol (MGDG) and phosphatidylglycerol (PG) in the chloroplast outer membrane. Whether they are inserted by the TOC complex or an undiscovered insertase is unknown, but interaction with the TOC complex is an essential step in their targeting. Chloroplast outer membrane (COM); intermembrane space (IMS); chloroplast inner membrane (CIM). Created using BioRender.com (accessed on 1 December 2021).

It is still an open question how the plant cell achieves selective targeting of SA proteins to chloroplasts and mitochondria. Despite the ambiguity in the targeting signals of chloroplast and mitochondrion SA proteins, a cytosolic factor has been identified that is responsible for targeting chloroplast SA proteins to the membrane surface. Ankyrin repeat-containing protein 2 (AKR2) interacts with the SA targeting signal during translation and acts as a chaperone to shield the hydrophobic TMD and prevent aggregation to keep the SA protein in a membrane insertion competent state [39,72,73]. AKR2 achieves selective targeting through its lipid binding domain, which recognizes monogalactosyldiacylglycerol, a lipid unique to plastid membranes, and phosphatidylglycerol headgroups [74]. AKR2 may recognize subtle differences in the density of positively charged residues and amino acid residue composition of the CPR as well as its distance from the TMD [27]. The mechanism by which SA proteins are inserted in the chloroplast outer membrane is not well understood, and there is some evidence that it may vary between SA proteins [27]. Currently, it is not clear whether SA proteins can insert spontaneously into the membrane or if a, yet to be discovered, insertase is involved; regardless, targeting and insertion seem to be dependent on the presence of the TOC complex [27]. The targeting mechanisms of SA, TA and β-barrel chloroplast outer membrane proteins are depicted in Figure 1.

### 4.2. Tail Anchored (TA) Proteins

Like SA proteins, TA proteins lack a cleavable TP, but are anchored in the membrane by a single α-helix at their C-terminus [75]. The TMD is flanked by a C-terminal sequence (CTS) and, together, the TMD and CTS act as an intrinsic targeting signal [76]. Interestingly, the physicochemical characteristics of the TA are not as important to targeting. The hydrophobicity of the TMD varies widely across TA proteins and the importance of the CTS varies from protein to protein, although a net positive charge seems to contribute to chloroplast-targeting specificity [39]. Like the CPR of SA proteins, this assists in the evasion of the SRP. Eliminating the net positive charge redirects TA proteins to the mitochondrion [37]. A subset of TA proteins contains an RK/ST motif in their CTS. This motif is interchangeable among TA proteins in the subset. The charge distribution within the RK/ST motif appears to play a larger role than net charge [69,77]. For some TA proteins, the CTS is both necessary and sufficient for targeting, where for others, it is necessary but not sufficient [37]. The latter seems to be the case for those TA proteins that contain GTPase (G) domains, such as the TOC34 receptors discussed in detail below. 

Like SA proteins, AKR2 also interacts with TA proteins in the cytosol to guide them to the chloroplast outer membrane, although there is evidence that other cytosolic factors are involved [37,69]. The targeting of TA proteins is well characterized in mammalian cells and yeast, and involves the guided entry of TA proteins (GET) and transmembrane recognition complex (TRC) pathways, respectively [78,79]. GET homologs have been identified in *Arabidopsis thaliana* [77] and have been implicated in the targeting of TOC34 receptors [80]. GET3B has been shown to target TA proteins to the thylakoid membrane [81]. Although cytosolic factors contribute to the efficiency of TA protein targeting, specificity for the chloroplast outer membrane seems to rely on lipid composition of the membrane, which may be important for insertion [82]. It was previously thought that SA and TA protein insertion in the chloroplast outer membrane occurred exclusively through interactions with the naked chloroplast outer membrane. More recently, it has been shown that targeting of both SA and TA proteins to the chloroplast outer membrane requires TOC75 and competes with precursor proteins for insertion, suggesting they do use the TOC complex, at least as a first step in their insertion [83].

## 5. Biogenesis of β-Barrel Chloroplast Outer Membrane Proteins

β-barrel proteins are characterized by their distinct topology from the more common α-helical TMDs of many membrane proteins. They are defined as proteins composed of 8–24 β-strands, where individual strands are usually 9–11 amino acids in length and are tilted approximately 45 degrees from the plane of the membrane. Alternating patterns of amino acid side chains result in amphiphilic segments with a hydrophilic face lining the interior of the pore and a hydrophobic face exposed to the lipid bilayer. The structure is stabilized by hydrogen bonding networks between the peptide backbone of neighbouring β-strands. These pores are found exclusively in the outer membrane of plastids and mitochondria of eukaryotes, as well as the outer membrane of Gram-negative bacteria from which they originated [84,85]. They act as membrane anchors or channels that recognize and transport a wide variety of substrates (ions, small molecules, peptides, nucleic acids, and proteins) with varying levels of specificity [84]. Their shared ancestry, along with their homologous structure and function would suggest easily identifiable targeting elements; but their primary sequences are highly divergent [27], complicating attempts to identify conserved targeting sequences. Their targeting information is thought to, instead, be dispersed among the primary sequence and displayed in the form of secondary and/or tertiary structures [38,86]. 

Bacterial and mitochondrial β-barrels contain β-signals, which are conserved motifs in the C-terminal β-strand(s) of the barrel [87] that initiate interactions with the β-barrel assembly machinery (BAM) and SAM complexes in bacterial and mitochondrial outer membranes, respectively. It was previously thought that membrane-embedded β-barrels were highly rigid and unlikely to open laterally. This is not the case. In Gram-negative bacteria and mitochondria, the β-signal acts as an insertion signal, triggering the lateral opening of BAM and SAM pores [88]. It has been shown that the targeting of β-barrel proteins to mitochondria relies on the hydrophobicity of the C-terminal β-hairpin. Specifically, a hydrophilic amino acid residue positioned at the C-terminus of the penultimate β-strand determines mitochondrial targeting. Interestingly, deletion of the C-terminal β-hairpin of chloroplast β-barrel proteins disrupts their targeting and redirects them to mitochondria [85]. Mislocalization of β-barrels between chloroplasts and mitochondria does not occur in plant cells but does occur in vitro [38], suggesting cytosolic factors yet to be discovered play a crucial role in targeting fidelity. Although mutagenesis can alter the localization of a chloroplast β-barrel to mitochondria [38], the reverse has not been demonstrated. This would suggest that chloroplast β-barrel proteins have gained additional targeting elements that both enable chloroplast localization and prevent localization to mitochondria. In fact, there is evidence that chloroplast β-barrels contain distinct groups of signals and may reach the chloroplast outer membrane using a variety of targeting pathways. Some may even be capable of self-insertion [89,90]. Unlike SA and TA proteins, the unassisted targeting of a β-barrel protein to the chloroplast outer membrane has not been demonstrated experimentally to date. For some chloroplast outer membrane β-barrels, targeting information is contained in a set of N-terminal β-strands that engage the TOC complex and trigger import into the IMS [91]. They are then integrated into the outer membrane by OEP80, a process dependent on the C-terminal β-strand [92]. OEP80 is required for the accumulation of other β-barrel proteins in the chloroplast outer membrane and may represent part of the machinery that recognizes and inserts β-barrels in the chloroplast outer membrane [93,94].

## 6. Targeting and Assembly of the TOC Complex

### 6.1. TOC75 Translocation Channel Targeting

**Figure 2:**
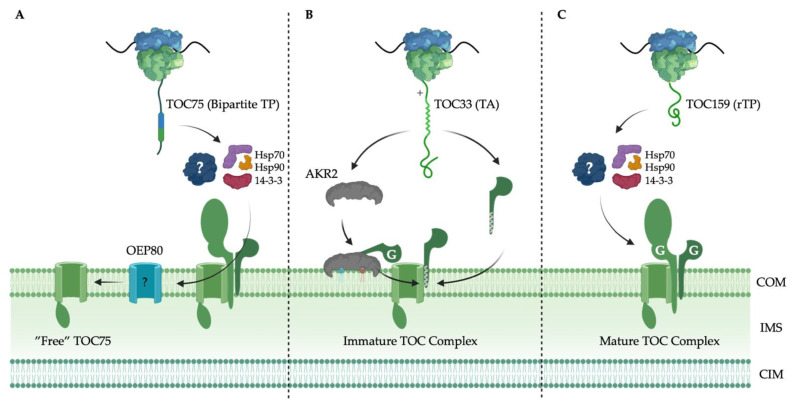
Targeting and assembly of the TOC complex: (**A**) TOC75 is targeted to the chloroplast outer membrane by a bipartite transit peptide (TP). The recognition and insertion of “free” TOC75 requires mature translocons at the outer membrane of the chloroplast (TOC complexes) and may be aided by OEP80. The presence of a TP suggests the use of chaperones such as Hsp70, Hsp90 and 14-3-3 employed by the canonical TP-mediated targeting pathway, although the existence of an uncharacterized cytosolic factor is possible; (**B**) TOC33/34 is a tail anchored (TA) protein and is targeted to the chloroplast outer membrane by both the TA and the GTPase (G) domain to form immature TOC complexes. Like other TA proteins, ankyrin repeat-containing protein 2 (AKR2) acts as a cytosolic chaperone; (**C**) TOC159/132/120 is targeted to the chloroplast outer membrane by a reverse TP-like sequence at the C-terminus (rTP), which may also engage chaperones employed by the canonical TP-mediated targeting pathway. Both TOC GTPase receptors rely on their G domains for successful targeting and TOC complex integration. The targeting of each component in sequence is coupled to the formation of mature TOC complexes. Chloroplast outer membrane (COM); intermembrane space (IMS); chloroplast inner membrane (CIM). Created using BioRender.com (accessed on 1 December 2021).

Although the components of the TOC complex fit well into the above-mentioned chloroplast outer membrane protein categories based on their structure and topology, their targeting signals and pathways seem to diverge from those described for β-barrel and TA proteins (Figure 2). TOC75 is the first component of the TOC complex to be inserted in the membrane and facilitates the targeting and insertion of TOC34 and TOC159 receptors [26,95,96]. TOC75 is a β-barrel protein, related to the OMP85 family of proteins that include the BAM and SAM β-barrels [97,98], although no other homologs of the Bam and SAM complex machinery have been detected in the chloroplast outer membrane. Interestingly, and unlike other β-barrel proteins, TOC75 is synthesized as a precursor protein in the cytosol and contains a cleavable bipartite N-terminal chloroplast TP [95,99]. The TP may help to ensure TOC75 targeting, and insertion is coupled to the formation of new TOC complexes, as well as to maintain its reverse topology [26]. Although there is evidence that existing TOC complexes facilitate the biogenesis of new TOC complexes in the chloroplast outer membrane [99], the bipartite signal could act as a temporary anchor to couple insertion by a β-barrel assembly machinery such as OEP80 with TOC complex integration [26]. A poly-glycine stretch downstream of the TP arrests import through the TOC-TIC super-complex and the TP and poly-glycine stretch are cleaved by the stromal processing peptidase and a type I signal peptidase in the IMS, respectively, yielding mature TOC75 that is inserted into the membrane by an unknown mechanism [99]. The poly-glycine stretch may also act to avoid proteinaceous factors in the IMS that drive import [100], instead releasing the protein to be inserted into the membrane laterally by the TOC complex, or via OEP80. Interestingly, OEP80 has been shown to contain a cleavable N-terminal TP, but not a poly-glycine stretch [101,102]. It is not isolated from mature TOC complexes [103] and its targeting is not in competition with chloroplast precursor proteins [101], suggesting it is inserted in a different manner than TOC75.

### 6.2. TOC34 GTPase Receptor Family Targeting 

“Free” TOC75, or TOC75 not associated with mature TOC complexes, serves as the site for TOC34 receptor integration in the chloroplast outer membrane and its assembly into maturing TOC complexes, simultaneously [96]. TOC34 receptors are TA proteins and are guided to the chloroplast outer membrane by interactions between their C-terminal α-helical TMD and AKR2 [37,104]. Arsenite/tail-anchored protein-transporting ATPase (ARSA1) has also been implicated in the targeting of TOC34 to the chloroplast outer membrane and TOM7, a mitochondrial TA protein, to mitochondria [78]. ARSA1 is similar to the GET proteins that target TA proteins to the ER in eukaryotes. Several ARSA homologs have been identified in plants and may be responsible for the targeting of TA proteins in plant cells [79]. There is evidence to support that TA proteins, like TOC34, engage the TOC complex thereafter [37]. Whether this occurs after insertion by an undiscovered insertase is unclear. Alternatively, there is evidence that interactions with galactolipids unique to the plastid membrane play an important role in TOC34 targeting [37]. This selectivity occurs at the membrane surface, independent of proteinaceous factors in the cytosol [82], and may facilitate self-insertion before or in addition to interaction with TOC75. Although there is evidence to suggest that TOC34 may self-insert into the chloroplast outer membrane [105,106,107], there is additional evidence to suggest that insertion is enhanced in the presence of nucleotides and inhibited when exposed to chloroplasts after proteolytic treatment [108], strengthening the theory that a yet-to-be-discovered insertase is involved. Additionally, the G domain of TOC34 receptors and the C-terminal tail exposed to the IMS have been implicated in targeting and integration into TOC complexes [106,109]. GTP hydrolysis could induce interactions between TOC34 monomers to produce TOC34 dimers that promote membrane insertion and/or TOC complex assembly [110]. Alternatively, GTP hydrolysis could induce an active conformation for monomeric TOC34 interaction with TOC75.

### 6.3. TOC159 GTPase Receptor Family Targeting

Relative to TOC75 and TOC34, we know very little about how TOC159 is targeted to and inserted in the chloroplast outer membrane [111]. Like TOC34, TOC159 targeting and insertion relies on TOC75, and there is evidence that TOC34 also supports the targeting and integration of TOC159 [107,112,113]. Whether the binding of TOC34 to TOC75 induces a conformation favourable to TOC159 binding and insertion and/or the interactions between the homologous G domains of the TOC34 and TOC159 receptors enhances these interactions, the membrane (M) domain clearly plays a critical role [5]. Several lines of evidence exist to support the targeting capability of the M domain [107,114,115]. The C-terminus of the M domain has demonstrated the ability to target fluorescent fusion proteins to the chloroplast outer membrane, likely due to a reverse TP-like sequence that shares physicochemical characteristics with canonical N-terminal chloroplast TPs [116,117]. This reverse TP may engage the other TOC complex components in the same way that chloroplast TPs do. Until recently, it was thought that TOC159 receptors used an atypical membrane anchor [118,119]. Recent structural prediction by AlphaFold would suggest that TOC159 receptors are anchored in the membrane by a β-barrel such as TOC75 [120]. The G domain also demonstrates intrinsic targeting capabilities [113], suggesting it may play an important role in targeting specificity and/or assembly of TOC159 into premature TOC complexes that contain TOC75 and TOC34 receptors [107]. In fact, it may even be required for the integration of TOC159 into mature TOC complexes [112,113]. It is important to distinguish between targeting, insertion, and TOC complex integration as they may represent distinct but interdependent processes. The targeting of TOC complex proteins likely evolved redundant targeting measures in this way to couple their targeting with the assembly of TOC complexes, further ensuring fidelity.

## 7. A Bioinformatic Approach to Identifying Novel Chloroplast Outer Membrane Targeting Signals and Pathways

**Figure 3:**
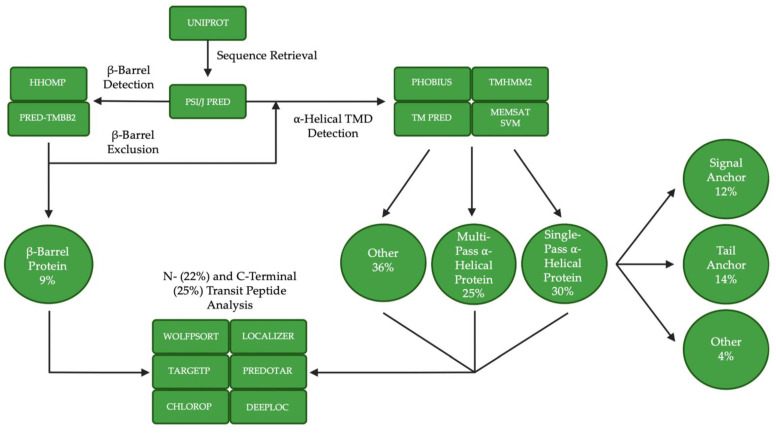
Bioinformatic approach to categorizing the chloroplast outer membrane proteome by targeting pathway. Bioinformatic tools used for sequence retrieval, β-barrel detection, β-barrel exclusion, α-helical transmembrane domain (TMD) detection and N- and C-terminal transit peptide detection [137,138,139,140,141,142,143,144,145,146,147,148] are provided in green squares, where resultant targeting pathway categories and percent composition of the proteome sorted into each category are given in green circles.

Much remains unknown about how most chloroplast outer membrane proteins make their way to and are inserted in the chloroplast outer membrane. The targeting of TOC complex components highlights the complexity of chloroplast outer membrane targeting pathways and their dependence on not only protein structure but coupled assembly into protein complexes. The fact that there are 50 proteins known to be dually targeted to chloroplasts and mitochondria further illustrates the subtle yet powerful role physicochemical characteristics within targeting signals play in targeting fidelity [121,122,123,124]. A better understanding of the variety of targeting signals within the chloroplast outer membrane proteome, the structures of their membrane anchors, physicochemical characteristics, and role in targeting and insertion is important in expanding our understanding of already characterized pathways and essential to identifying novel ones. We are the first to develop a bioinformatic approach (Figure 3) to categorize the chloroplast outer membrane proteome based on these properties. The goal of this approach is to select candidates for experimental studies that will be useful in the validation of additional targeting signals and pathways.

Briefly, we scanned the literature to produce an updated version of the chloroplast outer membrane proteome, generated by Inoue (2015) [30]. We identified 21 additional proteins with potential to localize to the chloroplast outer membrane [22,29], bringing the total number of proteins in the chloroplast outer membrane proteome to 138 (Table 1). We sorted these proteins into the functional categories provided by Inoue (2015) for the previous list of 117 proteins. Five proteins (At2g25660 [125], At3g49560 [126], At4g26670 [126], At5g24650 [126] and At5g55510 [127]) are involved in protein import; three proteins (At1g54150 [128], At1g59560 [128] and At5g13530 [129]) are involved in protein turnover and modification; five proteins (At2g40690 [130], At3g63520 [131], At4g12470 [132], At4g13550 [133] and At5g16010 [127]) are involved in lipid metabolism; one protein (At2g32290 [127]) is involved in carbohydrate metabolism and regulation; two proteins (At1g26340 [127] and At5g02580 [127]) are involved in other metabolism and regulation; two proteins (At2g34585 and At3g03870) have unknown functions; and three proteins (At3g07430 [134], At3g19720 [135] and At3g57090 [136]) are involved in organellar fission, a function not described by Inoue (2015). Next, we compiled a database that allowed us to group proteins based on structural and physicochemical characteristics (such as secondary structure elements, amino acid composition and pI at each terminus), experimentally validated to be unique to SA, TA and β-barrel proteins. The predictive aspects of the database were tested against experimentally determined structures and targeting function of well-studied chloroplast outer membrane proteins. Finally, we added elements to the database, which allowed us to identify potential chloroplast TPs at the N-terminus and reverse TP-like sequences at the C-terminus independent of categorized targeting pathways. At each stage, thresholds were established, and a small number of conflicts were investigated further by careful analysis of secondary structure predictions. Together, this approach allowed us to positively identify TOC75 and OEP80 as β-barrel proteins containing an N-terminal TP and TOC34 as a TA protein. The results showed that 30% of proteins were categorized as single pass α-helical proteins, with 12% being SA, 14% being TA and 4% being “other”; 25% of proteins were categorized as multi pass α-helical proteins; 9% of proteins were categorized as β-barrel proteins; and 36% of proteins were categorized as “other”, containing no predictable transmembrane elements. The 21 newly identified proteins are equally dispersed among the categories, except for the β-barrel category, into which none of the 21 newly identified proteins were sorted. Among the last group, 35% are predicted to contain a cleavable TP at their N-terminus and 39% are predicted to have a reverse TP-like sequence at their C-terminus. Interestingly, cleavable N-terminal TPs and reverse TP-like sequences at the C-terminus were also predicted, in small quantities, across all other categories, which suggests that more proteins than just TOC75 could use a bipartite signal sequence. This was also the case for the 21 newly identified chloroplast outer membrane proteins. It is also important to note that no protein was predicted to contain both an N-terminal TP and a reverse TP-like sequence at the C-Terminus. In summary, at least 65% of chloroplast outer membrane proteins were identified as having the potential to use novel targeting signals and pathways. Clearly, there is a disproportionate focus on traditional SA, TA and β-barrel-mediated targeting pathways in the literature.

## 8. Conclusions and Future Directions

In summary, plastids, such as the chloroplast, play a central role in a variety of metabolic and signalling processes within plant cells. The biogenesis and function of chloroplasts rely heavily on the fidelity of intracellular protein targeting pathways. Like mitochondria, chloroplasts evolved from an ancient bacterial endosymbiont and the two organelles share many common characteristics in the post-translational targeting of their nuclear-encoded proteomes [3]. Similar to mitochondrial presequences, chloroplast transit peptide sequences are highly divergent, but conserved physicochemical and structural properties govern their interactions with proteinaceous factors in the cytosol, recognition by import complexes at the membrane surface and even direct interactions with specific lipids [47], all of which contribute to targeting specificity. Despite recent advances in our understanding of the targeting of chloroplast precursor proteins and their recognition and import by the TOC and TIC complexes, much remains unknown about the targeting of chloroplast outer membrane proteins, such as TOC159. This gap is further emphasized when compared to our knowledge of outer membrane proteins of mitochondria. Here, we have reviewed the current understanding of how SA, TA and β-barrel chloroplast outer membrane proteins are targeted to the organelle. Further, we used a novel bioinformatic approach to expand the current list of known chloroplast outer membrane proteins from 117 to 138 and provided new insight into novel targeting signals and pathways that could be used by a significant portion of these proteins, yet to be explored experimentally. 

In mitochondria, two distinct complexes, SAM and MIM, ensure insertion and assembly of β-barrel and α-helical outer membrane proteins. These processes are mediated by the TOM complex [67]. Such complexes have not been identified in chloroplasts and so our understanding of the molecular mechanisms by which β-barrel and α-helical chloroplast outer membrane proteins are recognized, inserted and assembled at the chloroplast outer membrane is severely limited. Whether the TOC complex assumes these roles or, like the TOM complex, merely mediates interactions between membrane proteins and their insertases is unclear. It is crucial that future studies focus on identifying the targeting signals, cytosolic factors and integration complexes involved in chloroplast outer membrane protein biogenesis. Specifically, experiments should focus on clarifying the role of the TOC complex in the insertion of different types of chloroplast outer membrane proteins; identifying β-signals within the chloroplast outer membrane proteome and the cytosolic chaperone involved in β-barrel targeting; and further characterizing OEP80′s interaction with the TOC complex and its role in β-barrel insertion and assembly. Beyond this, we hope to further define bipartite signals that incorporate TPs and reverse TP-like sequences at the C-terminus, and how these sequences contribute to the modularity of targeting signals. AKR2 shuttles SA and TA proteins in the cytosol to the chloroplast outer membrane through specific interactions with lipids unique to the plastid membrane [74]. This emphasizes the interplay between the proteins of the outer membrane and the lipid molecules in which they are embedded; specifically, the role chloroplast outer membrane lipid composition plays in targeting specificity, another area that requires further exploration.

## Figures and Tables

**Table 1:** An updated list of the chloroplast outer membrane proteome and associated targeting pathways. Identified and predicted proteins of the chloroplast outer membrane proteome were categorized by potential targeting pathway and signal according to bioinformatic analyses.

AGI ^1^	Name ^2^	N TP ^3^	C TP-Like ^4^
**Signal Anchored (SA) Proteins**			
At1g67690	M3 Protease		✓
At2g19860	HXK2		
At2g34585 *	-		
At2g38670	PECT1		
At3g17970	TOC64-III		✓
At3g21865	PEX22		✓
At3g52420	OEP7		
At3g63170	FAP1	✓	
At4g12470 *	pEARLI1-Like Lipid Transfer Protein	✓	
At4g27680	NTPase		
At4g29130	HXK1		
At5g17770	CBR		
At5g20520	WAV2		✓
At5g25900	KO1/GA3		
At5g51020	CRL		
At5g64816	-		✓
**Tail Anchored (TA) Proteins**			
At1g02280	TOC33		
At1g09920	-		
At1g13900	PAP2		
At1g26340 *	Cytochrome b5		
At1g27300	-		
At1g27390	Tom20-2		
At2g16070	PDV2		
At2g32240	DUF869		
At3g03870 *	F20H23.8 Protein		✓
At3g27820	MDAR4		
At3g57090 *	Mitochondrial Fission 1 Protein		
At3g63150	MIRO2		
At4g32250	Tyrosine Kinase		
At4g35000	APX3		
At5g05000	TOC34		✓
At5g11560	-		✓
At5g21990	OEP61-TPR		
At5g27330	-		
At5g27540	MIRO1		
At5g56730	Peptidase M16		
**Other Single Pass** **α-Helical Proteins**			
At1g16000	OEP9		
At1g80890	OEP9.2		
At2g25660 *	TIC236	✓	✓
At3g52230	OMP24 Homolog		
At3g63160	OEP6		
**Multi-Pass** **α-Helical Proteins**			
At1g12230	Transaldolase	✓	
At1g34430	PDC E2	✓	
At1g44170	ALDH3H1		✓
At1g54150 *	E3 Ubiquitin-Protein Ligase SPL2		✓
At1g59560 *	E3 Ubiquitin-Protein Ligase SPL1		
At1g63900	SP1		✓
At1g64850	-		✓
At1g68680	-		
At1g77590	LACS9		✓
At2g01320	WBC7		
At2g11810	MGD3		✓
At2g28900	OEP16-1		
At2g34590	PDC E1 Beta	✓	
At2g40690 *	G3P Dehydrogenase	✓	
At2g44640	-		
At2g47770	TSPO		
At3g07430 *	YlmG Homolog	✓	
At3g49560 *	TIM Protein		
At3g51870	PAPST1 Homolog		
At3g62880	OEP16-4		
At4g15440	HPL Homolog		
At4g15810	NTPase		
At4g16160	OEP16-2		✓
At4g16450	Complex I Subunit		✓
At4g26670 *	TIM Protein		
At4g27990	YGGT-B Protein	✓	
At4g31780	MGD1	✓	
At4g38920	Vacuolar ATPase Subunit		
At5g06290	Prx B	✓	
At5g13530 *	E3 Ubiquitin-Protein Ligase KEG		
At5g16010 *	Dehydrogenase		
At5g21920	YGGT-A Protein		
At5g24650 *	TIM Protein		
At5g35210	PTM		
At5g55510 *	TIM Protein		
**β-Barrel Proteins**			
At1g20816	OEP21-1		
At1g45170	OEP24-1		
At1g76405	OEP21-2		
At2g06010	-		
At2g43950	OEP37	✓	
At3g01280	VDAC1		
At3g44160	P39/OEP80 TR1		
At3g46740	TOC75-III	✓	
At3g48620	P36/OEP80 TR2		
At4g09080	TOC75-IV		
At5g15090	VDAC3		
At5g19620	OEP80/TOC75-V	✓	
At5g42960	OEP24-2		
**Other Proteins**			
At1g02560	ClpP5	✓	
At1g07930	E-Tu		
At1g09340	CRB		
At1g70480	DUF220		✓
At2g16640	TOC132		✓
At2g17390	AKR2B		✓
At2g17695	OEP23		
At2g20890	THF1/PSB29	✓	
At2g24440	-		✓
At2g27490	ATCOAE		
At2g32290 *	Beta-Amylase 6	✓	
At2g32650	PTAC18-Like	✓	
At3g01500	Beta CA1	✓	
At3g06510	SFR2/GGGT		✓
At3g06960	TGD4		
At3g11670	DGD1	✓	
At3g12580	Hsc70-4		✓
At3g16620	TOC120		✓
At3g16950	PDC E3	✓	
At3g19720 *	ARC5		
At3g25690	CHUP1		
At3g25860	PDC E2	✓	
At3g26070	PAP/FBN3a	✓	
At3g26740	CCL	✓	
At3g46030	Histone H2B		✓
At3g46780	pTAC16	✓	
At3g49350	-	✓	
At3g53560	TPR Protein	✓	
At3g63520 *	Carotenoid Cleaving Protein		
At4g00550	DGD2		✓
At4g02482	Putative GTPase	✓	
At4g02510	TOC159		✓
At4g05050	UBQ11		
At4g13550 *	Putative Triglyceride Lipase	✓	
At4g14430	Enoyl-CoA Isomerase		✓
At4g17170	RAB2		✓
At4g36650	pBrP		
At5g02500	Hsc70-1		✓
At5g02580 *	Argininosuccinate Lyase		
At5g16870	PTH2 Family Protein		
At5g20300	TOC90		✓
At5g20410	MGD2		✓
At5g23190	CYP86B1		✓
At5g35360	CAC2/BC	✓	
At5g42070	-	✓	
At5g43070	WPP1		✓
At5g53280	PDV1		
At5g58140	PHOT2		✓
At5g59840	RAB8A-Like Protein		✓

^1^*Arabidopsis* gene identifier (AGI) number, ^2^ Proteins without a name are marked with -, ^3^ N-Terminal transit peptide (N TP), ^4^ TC-Terminal transit peptide-like sequence (C TP-Like), * Proteins not included in the list published by Inoue (2015) [30].

## References

[1] Gould S.B., Waller R.F., McFadden G.I. (2008). Plastid evolution. Annu. Rev. Plant Biol..

[2] Bölter B. (2018). En route into chloroplasts: Preproteins’ way home. Photosynth. Res..

[3] Day P.M., Theg S.M. (2018). Evolution of protein transport to the chloroplast envelope membranes. Photosynth. Res..

[4] Kessler F., Schnell D. (2009). Chloroplast biogenesis: Diversity and regulation of the protein import apparatus. Curr. Opin. Cell Biol..

[5] Paila Y.D., Richardson L.G.L., Schnell D.J. (2015). New insights into the mechanism of chloroplast protein import and its integration with protein quality control, organelle biogenesis and development. J. Mol. Biol..

[6] Choi H., Yi T., Ha S.H. (2021). Diversity of plastid types and their interconversions. Front. Plant Sci..

[7] Chu C.C., Swamy K., Li H.M. (2020). Tissue-specific regulation of plastid protein import via transit-peptide motifs. Plant Cell.

[8] Pogson B.J., Ganguly D., Albrecht-Borth V. (2015). Insights into chloroplast biogenesis and development. Biochim. Biophys. Acta-Bioenerg..

[9] Chu C.C., Li H.M. (2018). Developmental regulation of protein import into plastids. Photosynth. Res..

[10] Shanmugabalaji V., Chahtane H., Accossato S., Rahire M., Gouzerh G., Lopez-Molina L., Kessler F. (2018). Chloroplast biogenesis controlled by DELLA-TOC159 interaction in early plant development. Curr. Biol..

[11] Yan J., Smith M.D., Glick B.R., Liang Y. (2014). Effects of ACC deaminase containing rhizobacteria on plant growth and expression of Toc GTPases in tomato (*Solanum lycopersicum*) under salt stress. Botany.

[12] Doroodian P., Hua Z. (2021). The ubiquitin switch in plant stress response. Plants.

[13] Richardson L.G.L., Singhal R., Schnell D.J. (2017). The integration of chloroplast protein targeting with plant developmental and stress responses. BMC Biol..

[14] Jarvis P., López-Juez E. (2013). Biogenesis and homeostasis of chloroplasts and other plastids. Nat. Rev. Mol. Cell Biol..

[15] Yang X., Li Y., Qi M., Liu Y., Li T. (2019). Targeted control of chloroplast quality to improve plant acclimation: From protein import to degradation. Front. Plant Sci..

[16] Fukazawa H., Tada A., Richardson L.G.L., Kakizaki T., Uehara S., Ito-Inaba Y., Inaba T. (2020). Induction of *TOC* and *TIC* genes during photomorphogenesis is mediated primarily by cryptochrome 1 in Arabidopsis. Sci. Rep..

[17] Inoue H., Rounds C., Schnell D.J. (2010). The molecular basis for distinct pathways for protein import into arabidopsis chloroplasts. Plant Cell.

[18] Li H., Teng Y.S. (2013). Transit peptide design and plastid import regulation. Trends Plant Sci..

[19] Chu C.C., Li H.M. (2015). Protein import into isolated pea root leucoplasts. Front. Plant Sci..

[20] Leister D. (2003). Chloroplast research in the genomic age. Trends Genet..

[21] Kleffmann T., Russenberger D., Von Zychlinski A., Christopher W., Sjölander K., Gruissem W., Baginsky S. (2004). The *Arabidopsis thaliana* chloroplast proteome reveals pathway abundance and novel protein functions. Curr. Biol..

[22] Zybailov B., Rutschow H., Friso G., Rudella A., Emanuelsson O., Sun Q., Van Wijk K.J. (2008). Sorting signals, N-terminal modifications and abundance of the chloroplast proteome. PLoS ONE.

[23] Zimorski V., Ku C., Martin W.F., Gould S.B. (2014). Endosymbiotic theory for organelle origins. Curr. Opin. Microbiol..

[24] Jarvis P., Paul J. (2008). Targeting of nucleus-encoded proteins to chloroplasts in plants. New Phytol..

[25] Schnell D.J. (2019). The TOC GTPase receptors: Regulators of the fidelity, specificity and substrate profiles of the general protein import machinery of chloroplasts. Protein J..

[26] Richardson L.G.L., Paila Y.D., Siman S.R., Chen Y., Smith M.D., Schnell D.J. (2014). Targeting and assembly of components of the TOC protein import complex at the chloroplast outer envelope membrane. Front. Plant Sci..

[27] Kim J., Na Y.J., Park S.J., Baek S.H., Kim D.H. (2019). Biogenesis of chloroplast outer envelope membrane proteins. Plant Cell Rep..

[28] Sjuts I., Soll J., Bölter B. (2017). Import of soluble proteins into chloroplasts and potential regulatory mechanisms. Front. Plant Sci..

[29] Bouchnak I., Brugière S., Moyet L., Le Gall S., Salvi D., Kuntz M., Tardif M., Rolland N. (2019). Unraveling hidden components of the chloroplast envelope proteome: Opportunities and limits of better MS sensitivity. Mol. Cell. Proteom..

[30] Inoue K. (2015). Emerging knowledge of the organelle outer membranes—Research snapshots and an updated list of the chloroplast outer envelope proteins. Front. Plant Sci..

[31] Inoue K. (2011). Emerging roles of the chloroplast outer envelope membrane. Trends Plant Sci..

[32] Breuers F.K.H., Bräutigam A., Weber A.P.M. (2011). The plastid outer envelope—A highly dynamic interface between plastid and cytoplasm. Front. Plant Sci..

[33] Schleiff E., Tien R., Salomon M., Soll J. (2001). Lipid composition of outer leaflet of chloroplast outer envelope determines topology of OEP7. Mol. Biol. Cell.

[34] Cline K., Keegstra K. (1983). Galactosyltransferases involved in galactolipid biosynthesis are located in the outer membrane of pea chloroplast envelopes. Plant Physiol..

[35] Sahin C., Reid D.J., Marty M.T., Landreh M. (2020). Scratching the surface: Native mass spectrometry of peripheral membrane protein complexes. Biochem. Soc. Trans..

[36] Murcha M.W., Kmiec B., Kubiszewski-Jakubiak S., Teixeira P.F., Glaser E., Whelan J. (2014). Protein import into plant mitochondria: Signals, machinery, processing, and regulation. J. Exp. Bot..

[37] Dhanoa P.K., Richardson L.G.L., Smith M.D., Gidda S.K., Henderson M.P.A., Andrews D.W., Mullen R.T. (2010). Distinct pathways mediate the sorting of tail-anchored proteins to the plastid outer envelope. PLoS ONE.

[38] Lee D.W., Lee J., Hwang I. (2017). Sorting of nuclear-encoded chloroplast membrane proteins. Curr. Opin. Plant Biol..

[39] Lee J., Kim D.H., Hwang I. (2014). Specific targeting of proteins to outer envelope membranes of endosymbiotic organelles, chloroplasts, and mitochondria. Front. Plant Sci..

[40] Richardson L.G.L., Schnell D.J. (2020). Origins, function, and regulation of the TOC-TIC general protein import machinery of plastids. J. Exp. Bot..

[41] Thomson S.M., Pulido P., Jarvis R.P. (2020). Protein import into chloroplasts and its regulation by the ubiquitin-proteasome system. Biochem. Soc. Trans..

[42] Andrès C., Agne B., Kessler F. (2010). The TOC complex: Preprotein gateway to the chloroplast. Biochim. Biophys. Acta-Mol. Cell Res..

[43] Nakai M. (2018). New perspectives on chloroplast protein import. Plant Cell Physiol..

[44] Smith M.D. (2006). Protein import into chloroplasts: An ever-evolving story. Can. J. Bot..

[45] Lee D.W., Jung C., Hwang I. (2013). Cytosolic events involved in chloroplast protein targeting. Biochim. Biophys. Acta-Mol. Cell Res..

[46] Patron N.J., Waller R.F. (2007). Transit peptide diversity and divergence: A global analysis of plastid targeting signals. BioEssays.

[47] Lee D.W., Hwang I. (2018). Evolution and design principles of the diverse chloroplast transit peptides. Mol. Cells.

[48] Lee D.W., Yoo Y.J., Razzak M.A., Hwang I. (2018). Prolines in transit peptides are crucial for efficient preprotein translocation into chloroplasts. Plant Physiol..

[49] Bruce B.D. (2001). The paradox of plastid transit peptides: Conservation of function despite divergence in primary structure. Biochim. Biophys. Acta-Mol. Cell Res..

[50] Dong W.L., Jong K.K., Lee S., Choi S., Kim S., Hwang I. (2008). Arabidopsis nuclear-encoded plastid transit peptides contain multiple sequence subgroups with distinctive chloroplast-targeting sequence motifs. Plant Cell.

[51] Lee D.W., Lee S., Lee J., Woo S., Razzak M.A., Vitale A., Hwang I. (2019). Molecular mechanism of the specificity of protein import into chloroplasts and mitochondria in plant cells. Mol. Plant.

[52] Flores-Pérez Ú., Jarvis P. (2013). Molecular chaperone involvement in chloroplast protein import. Biochim. Biophys. Acta-Mol. Cell Res..

[53] Hristou A., Grimmer J., Baginsky S. (2020). The secret life of chloroplast precursor proteins in the cytosol. Mol. Plant.

[54] Panigrahi R., Whelan J., Vrielink A. (2014). Exploring ligand recognition, selectivity and dynamics of TPR domains of chloroplast Toc64 and mitochondria Om64 from *Arabidopsis thaliana*. J. Mol. Recognit..

[55] Qbadou S., Becker T., Mirus O., Tews I., Soll J., Schleiff E. (2006). The molecular chaperone Hsp90 delivers precursor proteins to the chloroplast import receptor Toc64. EMBO J..

[56] Kouranov A., Schnell D.J. (1997). Analysis of the interactions of preproteins with the import machinery over the course of protein import into chloroplasts. J. Cell Biol..

[57] Smith M.D., Rounds C.M., Wang F., Chen K., Afitlhile M., Schnell D.J. (2004). atToc159 is a selective transit peptide receptor for the import of nucleus-encoded chloroplast proteins. J. Cell Biol..

[58] Kessler F., Blobel G., Patel H.A., Schnell D.J. (1994). Identification of two GTP-binding proteins in the chloroplast protein import machinery. Science.

[59] Yan J., Campbell J.H., Glick B.R., Smith M.D., Liang Y. (2014). Molecular characterization and expression analysis of chloroplast protein import components in tomato (*Solanum lycopersicum*). PLoS ONE.

[60] Ramundo S., Asakura Y., Salomé P.A., Strenkert D., Boone M., Mackinder L.C.M., Takafuji K., Dinc E., Rahire M., Crèvecoeur M. (2020). Coexpressed subunits of dual genetic origin define a conserved supercomplex mediating essential protein import into chloroplasts. Proc. Natl. Acad. Sci. USA.

[61] Agne B., Kessler F. (2009). Protein transport in organelles: The Toc complex way of preprotein import. FEBS J..

[62] Schleiff E., Soll J., Küchler M., Kühlbrandt W., Harrer R. (2003). Characterization of the translocon of the outer envelope of chloroplasts. J. Cell Biol..

[63] Chen L.J., Li H.M. (2017). Stable megadalton TOC–TIC supercomplexes as major mediators of protein import into chloroplasts. Plant J..

[64] Kikuchi S., Hirohashi T., Nakai M. (2006). Characterization of the preprotein translocon at the outer envelope membrane of chloroplasts by blue native PAGE. Plant Cell Physiol..

[65] Chen K.Y., Li H.M. (2007). Precursor binding to an 880-kDa Toc complex as an early step during active import of protein into chloroplasts. Plant J..

[66] Jackson-Constan D., Keegstra K. (2001). Arabidopsis genes encoding components of the chloroplastic protein import apparatus. Plant Physiol..

[67] Gupta A., Becker T. (2021). Mechanisms and pathways of mitochondrial outer membrane protein biogenesis. Biochim. Biophys. Acta-Bioenerg..

[68] Lundquist K., Billings E., Bi M., Wellnitz J., Noinaj N. (2021). The assembly of β-barrel membrane proteins by BAM and SAM. Mol. Microbiol..

[69] Teresinski H.J., Gidda S.K., Nguyen T.N.D., Howard N.J.M., Porter B.K., Grimberg N., Smith M.D., Andrews D.W., Dyer J.M., Mullen R.T. (2019). An RK/ST C-terminal motif is required for Ttrgeting of OEP7.2 and a subset of other Arabidopsis tail-anchored proteins to the plastid outer envelope membrane. Plant Cell Physiol..

[70] Lee J., Lee H., Kim J., Lee S., Kim D.H., Kim S., Hwang I. (2011). Both the hydrophobicity and a positively charged region flanking the c-terminal region of the transmembrane domain of signal-anchored proteins play critical roles in determining their targeting specificity to the endoplasmic reticulum or endosymbiotic org. Plant Cell.

[71] Lee Y.J., Sohn E.J., Lee K.H., Lee D.W., Hwang I. (2004). The transmembrane domain of AtTco64 and its C-terminal lysine-rich flanking region are targeting signals to the chloroplast outer envelope membrane. Mol. Cells.

[72] Bae W., Lee Y.J., Kim D.H., Lee J., Kim S., Sohn E.J., Hwang I. (2008). AKR2A-mediated import of chloroplast outer membrane proteins is essential for chloroplast biogenesis. Nat. Cell Biol..

[73] Kim D.H., Lee J.E., Xu Z.Y., Geem K.R., Kwon Y., Park J.W., Hwang I. (2015). Cytosolic targeting factor AKR2A captures chloroplast outer membrane-localized client proteins at the ribosome during translation. Nat. Commun..

[74] Kim D.H., Park M.J., Gwon G.H., Silkov A., Xu Z.Y., Yang E.C., Song S., Song K., Kim Y., Yoon H.S. (2014). An ankyrin repeat domain of AKR2 drives chloroplast targeting through coincident binding of two chloroplast lipids. Dev. Cell.

[75] Zhuang X., Chung K.P., Jiang L. (2017). Targeting tail-anchored proteins into plant organelles. Proc. Natl. Acad. Sci. USA.

[76] Moog D. (2019). Higher complexity requires higher accuracy: Tail-anchored protein targeting to the outer envelope membrane of plant plastids via a specific C-terminal motif. Plant Cell Physiol..

[77] Marty N.J., Teresinski H.J., Hwang Y.T., Clendening E.A., Gidda S.K., Sliwinska E., Zhang D., Miernyk J.A., Brito G.C., Andrews D.W. (2014). New insights into the targeting of a subset of tail-anchored proteins to the outer mitochondrial membrane. Front. Plant Sci..

[78] Formighieri C., Cazzaniga S., Kuras R., Bassi R. (2013). Biogenesis of photosynthetic complexes in the chloroplast of Chlamydomonas reinhardtii requires ARSA1, a homolog of prokaryotic arsenite transporter and eukaryotic TRC40 for guided entry of tail-anchored proteins. Plant J..

[79] Lin T.W., Chen C.C., Wu S.M., Chang Y.C., Li Y.C., Su Y.W., Hsiao C.D., Chang H.Y. (2019). Structural analysis of chloroplast tail-anchored membrane protein recognition by ArsA1. Plant J..

[80] Maestre-Reyna M., Wu S.M., Chang Y.C., Chen C.C., Maestre-Reyna A., Wang A.H.J., Chang H.Y. (2017). In search of tail-anchored protein machinery in plants: Reevaluating the role of arsenite transporters. Sci. Rep..

[81] Anderson S.A., Satyanarayan M.B., Wessendorf R.L., Lu Y., Fernandez D.E. (2021). A homolog of GuidedEntry of Tail-anchored proteins3 functions in membrane-specific protein targeting in chloroplasts of Arabidopsis. Plant Cell.

[82] Kriechbaumer V., Abell B.M. (2012). Chloroplast envelope protein targeting fidelity is independent of cytosolic components in dual organelle assays. Front. Plant Sci..

[83] Tu S.L., Chen L.J., Smith M.D., Su Y.S., Schnell D.J., Li H.M. (2004). Import pathways of chloroplast interior proteins and the outer-membrane protein OEP14 converge at Toc75. Plant Cell.

[84] Jores T., Rapaport D. (2017). Early stages in the biogenesis of eukaryotic β-barrel proteins. FEBS Lett..

[85] Klinger A., Gosch V., Bodensohn U., Ladig R., Schleiff E. (2019). The signal distinguishing between targeting of outer membrane β-barrel protein to plastids and mitochondria in plants. Biochim. Biophys. Acta-Mol. Cell Res..

[86] Walther D.M., Rapaport D. (2009). Biogenesis of mitochondrial outer membrane proteins. Biochim. Biophys. Acta-Mol. Cell Res..

[87] Jores T., Klinger A., Groß L.E., Kawano S., Flinner N., Duchardt-Ferner E., Wöhnert J., Kalbacher H., Endo T., Schleiff E. (2016). Characterization of the targeting signal in mitochondrial β-barrel proteins. Nat. Commun..

[88] Höhr A.I.C., Lindau C., Wirth C., Qiu J., Stroud D.A., Kutik S., Guiard B., Hunte C., Becker T., Pfanner N. (2018). Membrane protein insertion through a mitochondrial β-barrel gate. Science.

[89] Horne J.E., Brockwell D.J., Radford S.E. (2020). Role of the lipid bilayer in outer membrane protein folding in Gram-negative bacteria. J. Biol. Chem..

[90] Gessmann D., Chung Y.H., Danoff E.J., Plummer A.M., Sandlin C.W., Zaccai N.R., Fleming K.G. (2014). Outer membrane β-barrel protein folding is physically controlled by periplasmic lipid head groups and BamA. Proc. Natl. Acad. Sci. USA.

[91] Day P.M., Inoue K., Theg S.M. (2019). Chloroplast outer membrane b-barrel proteins use components of the general import apparatus. Plant Cell.

[92] Gross L.E., Klinger A., Spies N., Ernst T., Flinner N., Simm S., Ladig R., Bodensohn U., Schleiff E. (2021). Insertion of plastidic b-barrel proteins into the outer envelopes of plastids involves an intermembrane space intermediate formed with Toc75-V/OEP80. Plant Cell.

[93] Huang W., Ling Q., Bédard J., Lilley K., Jarvis P. (2011). In vivo analyses of the roles of essential Omp85-related proteins in the chloroplast outer envelope membrane. Plant Physiol..

[94] Patel R., Hsu S.C., Bédard J., Inoue K., Jarvis P. (2008). The Omp85-related chloroplast outer envelope protein OEP80 is essential for viability in Arabidopsis. Plant Physiol..

[95] Tranel P.J., Keegstra K. (1996). A novel, bipartite transit peptide targets OEP75 to the outer membrane of the chloroplastic envelope. Plant Cell.

[96] Kouranov A., Chen X., Fuks B., Schnell D.J. (1998). Tic20 and Tic22 are new components of the protein import apparatus at the chloroplast inner envelope membrane. J. Cell Biol..

[97] Day P.M., Potter D., Inoue K. (2014). Evolution and targeting of omp85 homologs in the chloroplast outer envelope membrane. Front. Plant Sci..

[98] Schleiff E., Becker T. (2011). Common ground for protein translocation: Access control for mitochondria and chloroplasts. Nat. Rev. Mol. Cell Biol..

[99] Inoue K., Baldwin A.J., Shipman R.L., Matsui K., Theg S.M., Ohme-Takagi M. (2005). Complete maturation of the plastid protein translocation channel requires a type I signal peptidase. J. Cell Biol..

[100] Endow J.K., Rocha A.G., Baldwin A.J., Roston R.L., Yamaguchi T., Kamikubo H., Inoue K. (2016). Polyglycine acts as a rejection signal for protein transport at the chloroplast envelope. PLoS ONE.

[101] Inoue K., Potter D. (2004). The chloroplastic protein translocation channel Toc75 and its paralog OEP80 represent two distinct protein families and are targeted to the chloroplastic outer envelope by different mechanisms. Plant J..

[102] Gross L.E., Spies N., Simm S., Schleiff E. (2020). Toc75-V/OEP80 is processed during translocation into chloroplasts, and the membrane-embedded form exposes its POTRA domain to the intermembrane space. FEBS Open Bio.

[103] Eckart K., Eichacker L., Sohrt K., Schleiff E., Heins L., Soll J. (2002). A Toc75-like protein import channel is abundant in chloroplasts. EMBO Rep..

[104] Kim D.H., Hwang I. (2013). Direct targeting of proteins from the cytosol to organelles: The ER versus endosymbiotic organelles. Traffic.

[105] Schleiff E., Klösgen R.B. (2001). Without a little help from “my” friends: Direct insertion of proteins into chloroplast membranes?. Biochim. Biophys. Acta-Mol. Cell Res..

[106] Qbadou S., Tien R., Soll J., Schleiff E. (2003). Membrane insertion of the chloroplast outer envelope protein, Toc34: Constrains for insertion and topology. J. Cell Sci..

[107] Wallas T.R., Smith M.D., Sanchez-Nieto S., Schnell D.J. (2003). The roles of Toc34 and Toc75 in targeting the Toc159 preprotein receptor to chloroplasts. J. Biol. Chem..

[108] Hofmann N.R., Theg S.M. (2005). Chloroplast outer membrane protein targeting and insertion. Trends Plant Sci..

[109] Sun Y.J., Forouhar F., Li H.M., Tu S.L., Yeh Y.H., Kao S., Shr H.L., Chou C.C., Chen C., Hsiao C.D. (2002). Crystal structure of pea toc34, a novel gtpase of the chloroplast protein translocon. Nat. Struct. Biol..

[110] Lee J., Wang F., Schnell D.J. (2009). Toc receptor dimerization participates in the initiation of membrane translocation during protein import into chloroplasts. J. Biol. Chem..

[111] Ivanova Y., Smith M.D., Chen K., Schnell D.J. (2004). Members of the Toc159 import receptor family represent distinct pathways for protein targeting to plastids. Mol. Biol. Cell.

[112] Smith M.D., Hiltbrunner A., Kessler F., Schnell D.J. (2002). The targeting of the atToc159 preprotein receptor to the chloroplast outer membrane is mediated by its GTPase domain and is regulated by GTP. J. Cell Biol..

[113] Bauer J., Hiltbrunner A., Weibel P., Vidi P.A., Alvarez-Huerta M., Smith M.D., Schnell D.J., Kessler F. (2002). Essential role of the G-domain in targeting of the protein import receptor atToc159 to the chloroplast outer membrane. J. Cell Biol..

[114] Muckel E., Soll J. (1996). A protein import receptor of chloroplasts is inserted into the outer envelope membrane by a novel pathway. J. Biol. Chem..

[115] Lee K.H., Kim S.J., Lee Y.J., Jin J.B., Hwang I. (2003). The M domain of atToc159 plays an essential role in the import of proteins into chloroplasts and chloroplast biogenesis. J. Biol. Chem..

[116] Lung S.C., Smith M.D., Weston J.K., Gwynne W., Secord N., Chuong S.D.X. (2014). The C-terminus of Bienertia sinuspersici Toc159 contains essential elements for its targeting and anchorage to the chloroplast outer membrane. Front. Plant Sci..

[117] Lung S.C., Chuong S.D.X. (2012). A transit peptide-like sorting signal at the C terminus directs the bienertia sinuspersici preprotein receptor toc159 to the chloroplast outer membrane. Plant Cell.

[118] Bauer J., Chen K., Hiltbunner A., Wehrli E., Eugster M., Schnell D., Kessler F. (2000). The major protein import receptor of plastids is essential for chloroplast biogenesis. Nature.

[119] Chen K., Chen X., Schnell D.J. (2000). Initial binding of preproteins involving the Toc159 receptor can be bypassed during protein import into chloroplasts. Plant Physiol..

[120] Tunyasuvunakool K., Adler J., Wu Z., Green T., Zielinski M., Žídek A., Bridgland A., Cowie A., Meyer C., Laydon A. (2021). Highly accurate protein structure prediction for the human proteome. Nature.

[121] Sharma M., Bennewitz B., Klösgen R.B. (2018). Dual or not dual?—comparative analysis of fluorescence microscopy-based approaches to study organelle targeting specificity of nuclear-encoded plant proteins. Front. Plant Sci..

[122] Garg S.G., Gould S.B. (2016). The role of charge in protein targeting evolution. Trends Cell Biol..

[123] Ge C., Spånning E., Glaser E., Wieslander Å. (2014). Import determinants of organelle-specific and dual targeting peptides of mitochondria and chloroplasts in *Arabidopsis thaliana*. Mol. Plant.

[124] Carrie C., Small I. (2013). A reevaluation of dual-targeting of proteins to mitochondria and chloroplasts. Biochim. Biophys. Acta-Mol. Cell Res..

[125] Chen Y.L., Chen L.J., Chu C.C., Huang P.K., Wen J.R., Li H. (2018). min TIC236 links the outer and inner membrane translocons of the chloroplast. Nature.

[126] Rossig C., Reinbothe C., Gray J., Valdes O., Von Wettstein D., Reinbothe S. (2013). Three proteins mediate import of transit sequence-less precursors into the inner envelope of chloroplasts in *Arabidopsis thaliana*. Proc. Natl. Acad. Sci. USA.

[127] Gaudet P., Livstone M.S., Lewis S.E., Thomas P.D. (2011). Phylogenetic-based propagation of functional annotations within the Gene Ontology consortium. Brief. Bioinform..

[128] Ling Q., Jarvis P. (2015). Regulation of chloroplast protein import by the ubiquitin E3 ligase SP1 is important for stress tolerance in plants. Curr. Biol..

[129] Liu H., Stone S.L. (2010). Abscisic acid increases Arabidopsis ABI5 transcription factor levels by promoting KEG E3 ligase self-ubiquitination and proteasomal degradation. Plant Cell.

[130] Kachroo A., Venugopal S.C., Lapchyk L., Falcone D., Hildebrand D., Kachroo P. (2004). Oleic acid levels regulated by glycerolipid metabolism modulate defense gene expression in Arabidopsis. Proc. Natl. Acad. Sci. USA.

[131] Schwartz S.H., Qin X., Zeevaart J.A.D. (2001). Characterization of a Novel Carotenoid Cleavage Dioxygenase from Plants. J. Biol. Chem..

[132] Xu Z.Y., Zhang X., Schläppi M., Xu Z.Q. (2011). Cold-inducible expression of AZI1 and its function in improvement of freezing tolerance of *Arabidopsis thaliana* and Saccharomyces cerevisiae. J. Plant Physiol..

[133] Higashi Y., Okazaki Y., Takano K., Myouga F., Shinozaki K., Knoch E., Fukushima A., Saito K. (2018). HEAT INDUCIBLE LIPASE1 remodels chloroplastic monogalactosyldiacylglycerol by liberating α-linolenic acid in arabidopsis leaves under heat stress. Plant Cell.

[134] Kabeya Y., Nakanishi H., Suzuki K., Ichikawa T., Kondou Y., Matsui M., Miyagishima S. (2010). ya The YlmG protein has a conserved function related to the distribution of nucleoids in chloroplasts and cyanobacteria. BMC Plant Biol..

[135] Gao H., Kadirjan-Kalbach D., Froehlicht J.E., Osteryoung K.W. (2003). ARC5, a cytosolic dynamin-like protein from plants, is part of the chloroplast division machinery. Proc. Natl. Acad. Sci. USA.

[136] Zhang X., Hu J. (2009). Two small protein families, DYNAMIN-RELATED PROTEIN3 and FISSION1, are required for peroxisome fission in Arabidopsis. Plant J..

[137] Drozdetskiy A., Cole C., Procter J., Barton G.J. (2015). JPred4: A protein secondary structure prediction server. Nucleic Acids Res..

[138] Emanuelsson O., Nielsen H., Heijne G. (1999). Von ChloroP, a neural network-based method for predicting chloroplast transit peptides and their cleavage sites. Protein Sci..

[139] Krogh A., Larsson B., Von Heijne G., Sonnhammer E.L.L. (2001). Predicting transmembrane protein topology with a hidden Markov model: Application to complete genomes. J. Mol. Biol..

[140] Nugent T., Jones D.T. (2012). Detecting pore-lining regions in transmembrane protein sequences. BMC Bioinform..

[141] Bagos P.G., Liakopoulos T.D., Spyropoulos I.C., Hamodrakas S.J. (2004). PRED-TMBB: A web server for predicting the topology of β-barrel outer membrane proteins. Nucleic Acids Res..

[142] Käll L., Krogh A., Sonnhammer E.L.L. (2007). Advantages of combined transmembrane topology and signal peptide prediction-the Phobius web server. Nucleic Acids Res..

[143] Bateman A., Martin M.J., Orchard S., Magrane M., Agivetova R., Ahmad S., Alpi E., Bowler-Barnett E.H., Britto R., Bursteinas B. (2021). UniProt: The universal protein knowledgebase in 2021. Nucleic Acids Res..

[144] Horton P., Park K.J., Obayashi T., Fujita N., Harada H., Adams-Collier C.J., Nakai K. (2007). WoLF PSORT: Protein localization predictor. Nucleic Acids Res..

[145] Sperschneider J., Catanzariti A.M., Deboer K., Petre B., Gardiner D.M., Singh K.B., Dodds P.N., Taylor J.M. (2017). LOCALIZER: Subcellular localization prediction of both plant and effector proteins in the plant cell. Sci. Rep..

[146] Armenteros J.J.A., Salvatore M., Emanuelsson O., Winther O., Von Heijne G., Elofsson A., Nielsen H. (2019). Detecting sequence signals in targeting peptides using deep learning. Life Sci. Alliance.

[147] Small I., Peeters N., Legeai F., Lurin C. (2004). Predotar: A tool for rapidly screening proteomes for N-terminal targeting sequences. Proteomics.

[148] Almagro Armenteros J.J., Sønderby C.K., Sønderby S.K., Nielsen H., Winther O. (2017). DeepLoc: Prediction of protein subcellular localization using deep learning. Bioinformatics.

